# Non-traumatic Subdural Hematoma: A Rare Presentation of Cerebral Venous Sinus Thrombosis

**DOI:** 10.7759/cureus.103085

**Published:** 2026-02-06

**Authors:** Fouad Kaddour Hocine, Jessica Bauer, George S Zacharia, Muhammad Hammad Ashraf, Misbahuddin Khaja

**Affiliations:** 1 Internal Medicine, BronxCare Health System, New York, USA

**Keywords:** cerebral venous sinus, headache, subdural hematoma, thrombophilia, thrombosis

## Abstract

Cerebral venous sinus thrombosis (CVST) is rare and often overlooked, sometimes presenting as an unexplained subdural hematoma (SDH) without trauma. It very rarely accounts for a small proportion of all stroke cases, but carries significant morbidity and mortality if not promptly recognized and treated. We report the case of a middle-aged female who presented with a severe headache, initially diagnosed with SDH, with subsequent imaging revealing extensive dural venous sinus thrombosis. This case underscores the need to consider etiologies beyond conventional causes, including hypertension, trauma, and arterial aneurysm, in patients with intracerebral hemorrhage. Traditional non-contrast neuroimaging often fails to detect these rare thrombotic disorders. In contrast, contrast-enhanced imaging, especially with venography, promptly identifies the pathology. Anticoagulation remains the cornerstone of treatment for dural venous sinus thrombosis, as it prevents thrombus propagation and facilitates recanalization. Improved awareness, a high index of suspicion, and advanced imaging modalities are crucial for accurate diagnosis and, consequently, prompt management.

## Introduction

Cerebral venous sinus thrombosis (CVST) is an uncommon cerebrovascular disorder caused by thrombosis of the dural venous sinuses. The dural venous sinuses, also referred to as cortical venous sinuses, deliver venous blood from the brain to the systemic circulation. A blockade, typically seen with thrombosis, leads to impaired venous drainage and venous hypertension, with subsequent infarction or intracranial hemorrhage [1,2]. Multiple pathologies contribute to Virchow’s triad within this system, leading to thrombosis. Prothrombotic diseases, pregnancy and the puerperium, and oral contraceptive use are some of the best-described associations [1].

The superior sagittal sinus is most frequently involved in CVST [1]. Headache is the most frequent clinical presentation of CVST, reported by about 90% of symptomatic patients. In the absence of venous infarction or hemorrhage, focal neurological deficits are uncommon, except for sixth nerve palsy, which is a false localizing sign seen in patients with intracranial hypertension [1]. Unlike other forms of cerebrovascular disease, seizures are more common in patients with CVST, with reported frequencies as high as 40% [1]. CVST may present with non-traumatic subdural hematoma (SDH), thereby complicating the diagnostic process [3,4]. SDH associated with CVST is rare and may result from venous congestion and hypertension or from rupture of fragile bridging veins [5]. Magnetic resonance venography (MRV), with two-dimensional time-of-flight sequences, is the most frequently employed modality for diagnosis. A thrombophilia evaluation is recommended in all patients with CVST [1,2].

Anticoagulation is the first-line treatment in patients with CVST. For initial anticoagulation, unfractionated heparin or low-molecular-weight heparin is often preferred, with a subsequent transition to oral anticoagulants. Patients with unprovoked CVST require a longer duration of anticoagulation, while those with recurrent disease or underlying severe hypercoagulable conditions may require indefinite anticoagulation [1]. We report a case of CVST presenting as SDH in a middle-aged woman who presented with headache. We also provide a brief overview of the relevant literature concerning CVST, including diagnostic challenges and management.

## Case presentation

A 46-year-old female with a history of iron deficiency anemia presented to the emergency department with a three-day history of severe, holocranial headache, accompanied by vomiting and blurred vision. She denied any recent trauma, fever, syncope, seizures, or other sensory or motor symptoms. She reported not taking any regular medications, including contraceptive agents. She was obese and appeared uncomfortable due to the headache. She was hemodynamically stable, with a blood pressure of 138/74 mmHg. Neurological examination revealed no focal deficits or neck stiffness. Review of other systems was non-contributory. There were no skin rashes, bruising, petechiae, or purpura. 

**Figure 1:**
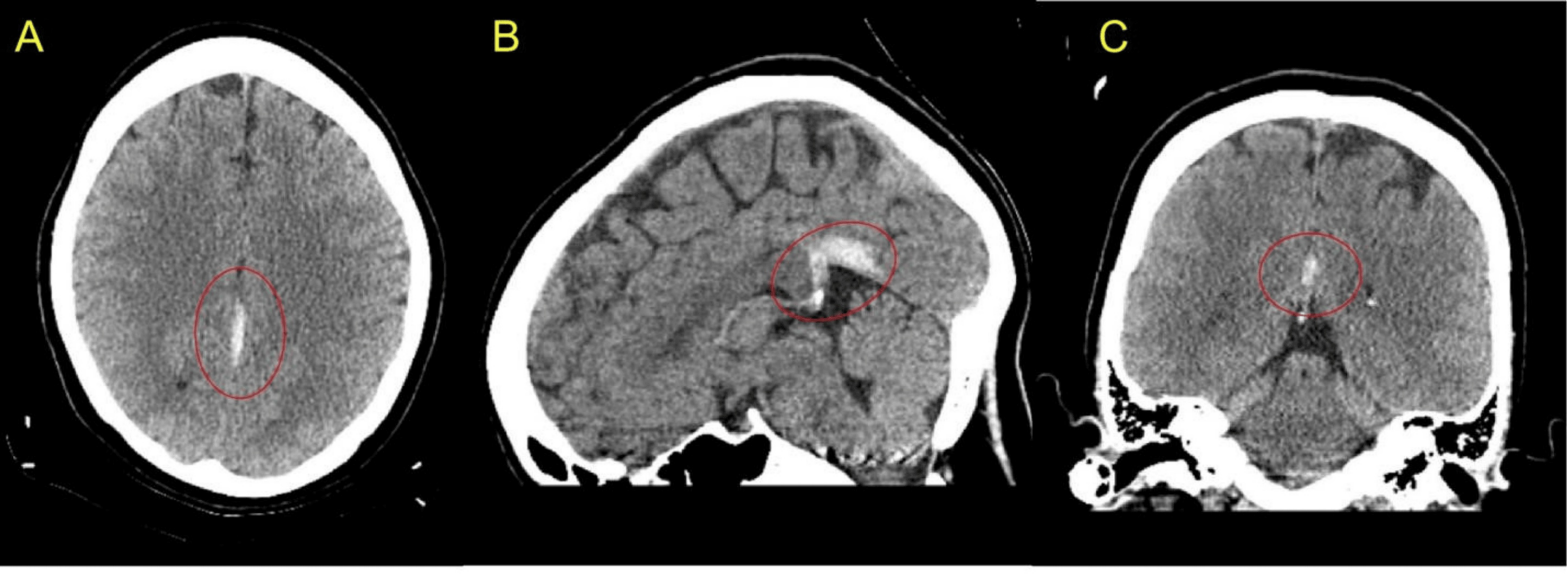
CT images Axial (A), sagittal (B), and coronal (C) images revealed a small acute subdural hematoma measuring approximately 3 mm along the left side of the falx cerebri, extending posteriorly along the left tentorium (encircled in red) CT: computed tomography

**Figure 2:**
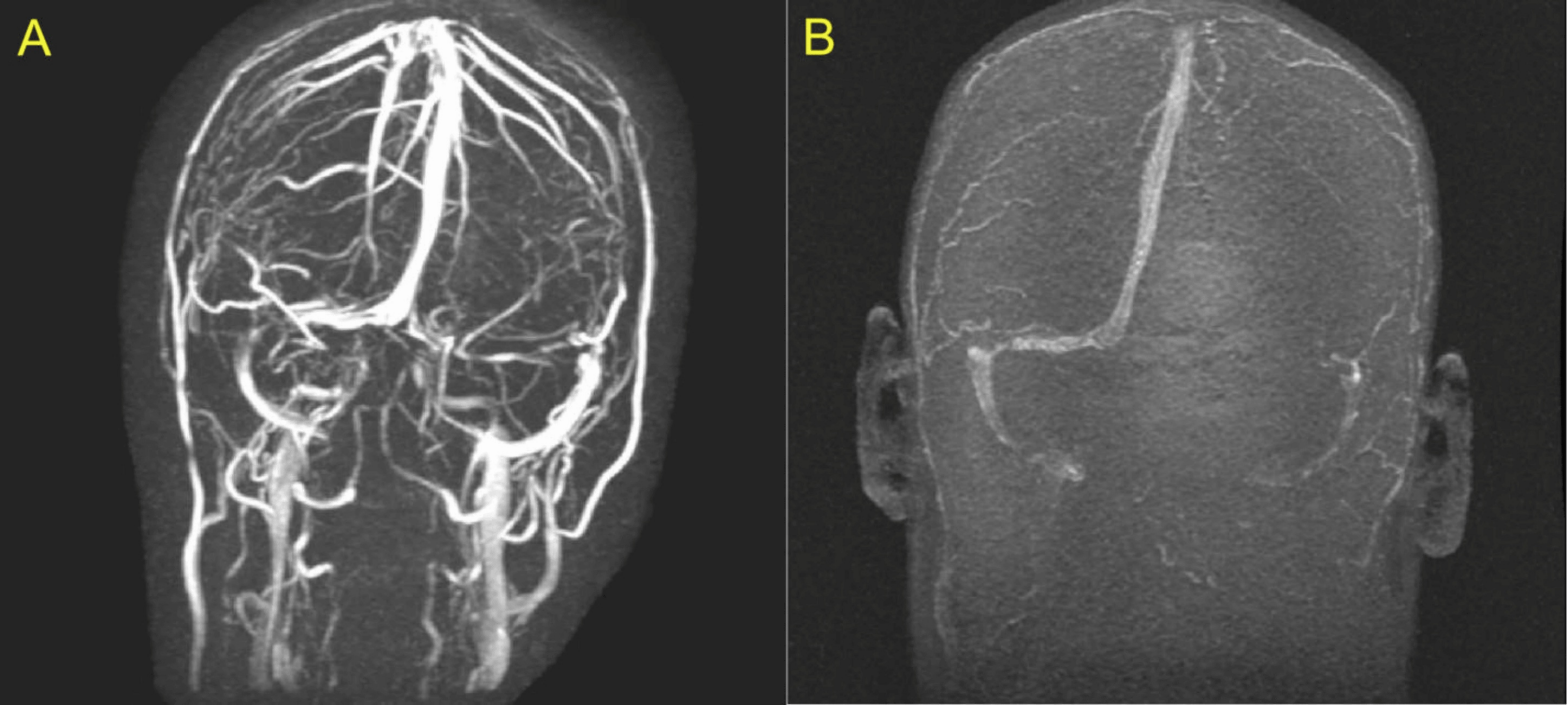
MRV images (A) 3-dimensional time of flight (3D-TOF) and (B) phase-contrast sequences demonstrated non-filling of the left transverse sinus, straight sinus, and proximal right sigmoid sinus, with partial filling of the distal right transverse sinus, concerning dural sinus thrombosis. The superior sagittal sinus was noted to be patent MRV: magnetic resonance venography

The initial laboratory evaluation was unremarkable, except for microcytic anemia (Table 1), later confirmed to be due to iron deficiency. Non-contrast CT of the head revealed a small SDH along the left falx and tentorium (Figure 1), with no evidence of mass effect. The patient was started on analgesics and antiemetics for symptomatic relief and admitted for close monitoring. Given the absence of trauma and the atypical location of the intracranial bleed, she underwent further evaluation with contrast-enhanced MRI of the head, including angiography and venography. Imaging revealed the SDH along with non-filling of the left transverse sinus, straight sinus, and proximal right sigmoid sinus, consistent with cerebral venous sinus thrombosis (CVST) (Figure 2). No aneurysms or vascular malformations were identified.

**Table 1:** Laboratory evaluation at presentation MCV: mean corpuscular volume; PT: prothrombin time; INR: international normalized ratio; aPTT: activated partial thromboplastin time; BUN: blood urea nitrogen; ALT: alanine aminotransferase; AST: aspartate aminotransferase; ALP: alkaline phosphatase

Parameter	Value	Reference range
Hemoglobin (g/dL)	9.1	12-16
MCV (fL)	66.7	80-96
Platelet count (×10³/µL)	468	150-400
PT (seconds)	11	10.4-15.7
INR	0.9	0.85-1.29
aPTT (seconds)	25.4	25-35
Creatinine (mg/dL)	0.8	0.5-1.5
BUN (mg/dL)	17	6-20
Bilirubin, total (mg/dL)	0.6	0.2-1.1
Bilirubin, direct (mg/dL)	0.3	0.1-0.2
ALT (IU/L)	28	5-40
AST (IU/L)	36	9-36
ALP (IU/L)	118	42-144
Ferritin (ng/mL)	15	18-300
Iron (mcg/dL)	25	60-170

Neurology and neurosurgery consultations were obtained for guidance on further evaluation and management. The patient was started on enoxaparin 40 mg subcutaneously with close monitoring. A repeat CT of the head at 24 hours showed no progression of the hemorrhage. She remained stable and reported symptomatic improvement with medical management. On the third day, low-molecular-weight heparin was transitioned to oral apixaban, 5 mg twice daily. She was discharged on apixaban and followed up in the neurology and hematology clinics. Extensive evaluation ruled out prothrombotic disorders (Table 2). At follow-up, the patient denied headache, nausea, visual disturbances, or bleeding complications except for menorrhagia, possibly related to anticoagulation. She remained neurologically stable and completed a total of three months of anticoagulation therapy.

**Table 2:** Summary of the thrombophilia workup Ig: immunoglobulin

Test	Result	Reference range
Lupus anticoagulant	Negative	Negative
Anti-cardiolipin antibodies (IgG, IgM, IgA)	Undetected	Undetected
Anti-phosphatidylserine antibodies (IgG, IgM)	Undetected	Undetected
Beta-2 glycoprotein I antibodies (IgG, IgM, IgA)	Undetected	Undetected
Antithrombin III activity	107%	80-135%
Protein C functional assay	145%	70-180%
Protein S functional assay	62%	60-140%
Factor V Leiden mutation	Negative	Negative
Total homocysteine	7.0 µmol/L	<10.0 µmol/L

## Discussion

Venous sinus thrombosis is a rare cause of non-traumatic intracranial hemorrhage, including SDH, which may result from venous hypertension and subsequent rupture of fragile bridging veins [1-5]. The estimated incidence of CVST in the United States is 2.62 per 100,000 hospitalized adults [6], accounting for 0.5% to 0.7% of all strokes [6]. Predisposing factors include age under 55 years, female sex, hypercoagulable or thrombophilic disorders, hormonal contraceptive or replacement therapy, obesity, malignancy, autoimmune diseases, medications, and trauma, whether iatrogenic or non-iatrogenic [1-9]. The superior sagittal sinus and transverse sinus are the most commonly affected sites in CVST [10].

Clinical features of CVST include headache, nausea, vomiting, seizures, blurred vision, diplopia, papilledema, encephalopathy, and coma [1]. Hemorrhage, including SDH, occurs in approximately 40% of patients. Around 10% present with severe symptoms such as thunderclap headache, subarachnoid hemorrhage, or acute focal neurologic deficits [1]. Diagnosis typically begins with a conventional CT or MRI. Direct visualization of the thrombus may be seen on initial non-contrast CT, where acute thrombus appears hyperdense (the "dense triangle sign" or "cord sign"), whereas on MRI, signal characteristics vary with thrombus age [1]. However, conventional CT is normal in approximately 70% of cases, making dedicated venous imaging essential [1]. The combination of MRI to visualize the thrombosed vessel and MRV to detect absent flow is considered the gold standard, as MRI alone can miss acute thrombosis due to flow artifacts and variable signal characteristics within the first three to five days [1,11].

Anticoagulation with low-molecular-weight heparin is preferred over unfractionated heparin, followed by transition to oral vitamin K antagonists or direct oral anticoagulants for 3-12 months in patients with transient risk factors, or indefinitely in those with chronic major thrombotic risk factors, and remains the cornerstone of CVST management [1], even in the presence of intracranial hemorrhage. It has been shown to improve outcomes by preventing thrombus propagation and promoting recanalization [12,13]. Endovascular therapy is reserved as a rescue option if neurological deterioration occurs despite adequate anticoagulation [1]. The American Society of Hematology recommends thrombophilia testing to guide treatment duration [1].

Our patient presented with chronic headache, previously misdiagnosed as migraine, and was diagnosed with SDH on initial CT of the head. The absence of trauma or other risk factors for intracranial hemorrhage, along with the atypical location of the bleed, prompted further evaluation, which led to the diagnosis of CVST. She had no overt risk factors for CVST except for her age and gender, and her prothrombotic work-up was negative. She was successfully treated with anticoagulation without complications, despite the presence of hemorrhage at presentation. This case highlights the importance of considering CVST in patients with unexplained chronic headache and non-traumatic SDH.

## Conclusions

Cerebral or dural venous sinus thrombosis is an uncommon cerebrovascular disorder. It is frequently associated with prothrombotic conditions, pregnancy or the puerperium, and hormonal contraceptive use, and typically presents with headache. Seizures are reported more often in patients with cerebral venous thrombosis than in those with conventional arterial stroke. Non-traumatic SDH, caused by venous hypertension or rupture of bridging veins, is an often-overlooked presentation of cortical venous sinus thrombosis. Maintaining a high index of suspicion and performing comprehensive neuroimaging allows for prompt diagnosis, with MRV being the preferred modality. Anticoagulation remains the treatment of choice in this patient population. Multidisciplinary care, preferably in stroke units, facilitates good outcomes in patients with cortical venous sinus thrombosis.
